# Epidemiology Informs Randomized Clinical Trials of Cognitive Impairments and Late-Onset, Sporadic Dementias

**DOI:** 10.29245/2572.942x/2018/5.1220

**Published:** 2018

**Authors:** Deborah R. Gustafson

**Affiliations:** 1 Department of Neurology, State University of New York, Downstate Medical Center, New York, USA; 2 Department of Health and Education, University of Skövde, Sweden

**Keywords:** Dementia, Epidemiology, Randomized clinical trials, Observational

Despite strong and consistent evidence^1^, criticisms of the association between vascular factors and cognitive impairments (CI) and late-onset, sporadic dementias (LOD), abound. A common critique is, “If vascular risk factors identified in observational epidemiology are real, why do clinical trials of treatments for vascular risk fail to improve cognition or prevent LOD?’ Concerns regarding shortfalls in statistical power, rigorous analyses, adjustment for multiple comparisons, precise exposure and outcome measurements, replication, and translational application, along with relatively low-risk estimates, flood the field. Despite this, stimulating scientific, innovative, critical, and controversial points of view persist because of observational epidemiology. Many forums for debate and platforms for biomarker development have been unveiled. The scientific community should not cast a bleak view of the vascular factor, LOD, life course, and aging epidemiology research landscape as what is too often portrayed and conveyed. From one vantage point, acknowledgment of these concerns provides a natural segue for the scientific community to focus on, facilitate and feed forward future solutions and fill knowledge gaps.

Given the strengths of Dementia Epidemiology in identifying population-level questions and solutions, how can there be a better translation to Randomized Controlled Trials (RCT) and precision medicine approaches? First and foremost, is an acknowledgment that Epidemiology, not RCT, more accurately reflects ‘real world’. The epidemiology embraces the variability, while an RCT seeks to control it. Other challenges include recognizing that: 1) CI and LOD result from exposures occurring over the life course; 2) definitions and terminology related to exposures and clinical outcomes are inconsistent; 3) characteristics of populations at risk vary; 4) mid-life, late-life associations are difficult to mimic in RCT; 5) there are a paucity of data on late life exposures and flux; 6) duration of exposure(s) (e.g., medication adherence) required to achieve desired effects observed in the observational epidemiology is ignored in most RCTs; 7) RCTs do not acknowledge and/or address reverse causality; and finally, typically ignored are 8) phenotypic changes with aging; 9) birth cohort; 10) multimorbidities; and 11) genetic susceptibility. RCT must embrace more of the chaos (intra- and inter-individual variabilities) to better identify prevention strategies that work (See Tables 1 and 2).

A theme among the aforementioned challenges is that life course epidemiology consistently points to the importance of timing (See Figure 1). Early-, mid- and late-life exposures in association with CI and LOD differ depending on when an exposure is measured and the length of time between exposure measurement and clinical disease onset. Early life exposures relate to developmental origins (in utero and neonatal development) hypotheses, in addition to educational attainment^2,3^. Mid-life exposures, typically measured between age 35–60 years, are most evolved for vascular (e.g., hypertension, overweight and obesity, hypercholesterolemia) and metabolic (e.g., Type 2 diabetes, adiposity, menopause) mechanisms; and burgeoning for chronic infectious diseases (e.g., Human Immunodeficiency Virus, HIV)^4,5^. Late-life exposures, typically measured at age 65 years and older in agreement with the age criterion for LOD, are dynamic, and reflect ageing-related changes, usually decline in mid-life vascular risk factors, such as declines in blood pressure^6^ and body weight^7–9^ from varying baseline levels that parallel or precede cognitive decline. This is underscored by comparing mid-versus late-life risk scores^10–13^. In addition, are the presence of multimorbidities, balanced or not by compensatory or resilience factors against a background of cognitive reserve^14^. In total, this life course milieu influences the manifestation of functional consequences and correlates of declining cognitive functions. In late-life, the timing of associations between vascular exposures and outcome is critical due to potential multi-stage brain and peripheral processes as well as the influence of evolving LOD-related neuropathologies on systemic ‘exposures’^15^. When measured in mid-life, higher levels of vascular risk factors are associated with higher LOD risk, however, when measured in late-life, vascular factors may be protective due to the influence of underlying LOD- or aging-related neuropathologies on their expression. This is termed ‘reverse causality’ and is observed in LOD association studies, with for example, body weight or body mass index (BMI), blood pressure and blood cholesterol levels. In addition, discovered in the 1990s, APOEε4 allele possession, encoding for a protein on the surface of lipoproteins, and influencing both lipid metabolism and LOD risk is an example of the major role of vascular risk factors in LOD etiology^16^. Other vascular susceptibility genes potentially modifying the effect of vascular phenotypes in association with LOD include for example, FTO and obesity^17^, ACE and blood pressure^18^, and clusterin/APOJ and blood cholesterol^19^.

Given this rich epidemiologic substrate, what are next steps to identify preventive agents for CI and LOD? The following example is one potential research question and study design that translates the epidemiology to the RCT.

## A Practical Example: The Case of Body Weight and Cognitive Impairments

Numerous observational epidemiology studies report on associations between adiposity (body weight, body mass index, waist circumference, waist-to-hip ratio, adipokines) and CI or LOD (See Table 4)^20–22^. Reverse causality and effect modification by APOEε4 is evident^8,20^. Metabolic dysregulation as measured via adipokines may inform^21^. Overweight and obesity are primary cardiovascular risk factors. Pharmacologic interventions for CI and Alzheimer’s disease (AD), and for aging-related chronic vascular diseases such as Type 2 diabetes, hypertension, and hyperlipidemias, induce side effects or phenotypic variations that are independent of their primary therapeutic indications. For example, there is sufficient literature to suggest that some treatments for CI (to include AD and related LOD) and aging vascular comorbidities cause body weight loss^23^. For example, acetylcholinesterase inhibitors (AChEI), at the forefront of symptomatic treatment of AD^24,25^, are associated with an almost 3-fold higher odds of body weight loss compared to placebo^26^; and medications used to treat Type 2 diabetes, for example, have differential effects on body weight gain and loss (See Table 3). While body weight loss or body weight gain may seem trivial, among aging adults, especially those experiencing clinical forms of CI or LOD, body weight loss and gain are not inconsequential and may cast the dice in favor of successful survival or death. Body weight loss may accelerate frailty, disability and death, while body weight gain or overweight and obesity may be beneficial^27^.

Provision of body weight gain therapies for CI and/or comorbidities among those with body weight loss, may maintain or improve cognitive function, prevent cognitive decline, and contribute to adequate peripheral health. While administration of certain drugs, alone or in combination, may create a ‘competing risk’ physiological environment that was not otherwise present. Combined therapies may become the front line treatment for patients with CI and are underweight or experiencing body weight loss, particularly among those possessing the APOEε4 allele.

An example study design is to compare patients with CI who are underweight or experiencing body weight loss with patients who are overweight or obese. This 18–24 month intervention is to compare commonly used AChEI, which as a class have been shown to be beneficial for cognition and contribute to body weight loss versus a combined therapy of AChEI plus a body weight enhancer, whether a medication for a comorbid condition that promotes body weight gain or a nutritional supplement (See Figure 2). Our primary hypothesis is that patients randomized to AChEI + body weight enhancer will maintain cognitive function and body weight, and have less cognitive decline, and behavioral and psychological symptoms, compared to those not receiving combined therapy. This hypothesis will be supported most strongly in the underweight or body weight loss group.

Our *secondary hypothesis* is that patients with at least one APOEe4 allele are especially susceptible to body weight loss and cognitive decline, whether they are underweight, overweight or obese, or experiencing body weight loss. Thus, they will respond less to AChEI alone or combined AChEI + body weight enhancer. Patients with at least one APOEe4 allele and receiving combination therapy will achieve better outcomes than those not receiving combined therapy, particularly those who are underweight or experiencing body weight loss. Measurement of adipokines, such as leptin may provide useful mechanistic insights.

Post hoc analyses would include stratification by the degree of body weight or BMI change prior to study initiation, consideration of smaller increments of baseline BMI, and increasing age group. Usual consideration of age, sex or gender, race/ethnicity, disparities, multimorbidities, and as other factors as aforementioned, apply.

## Conclusion

**Figure 1.**
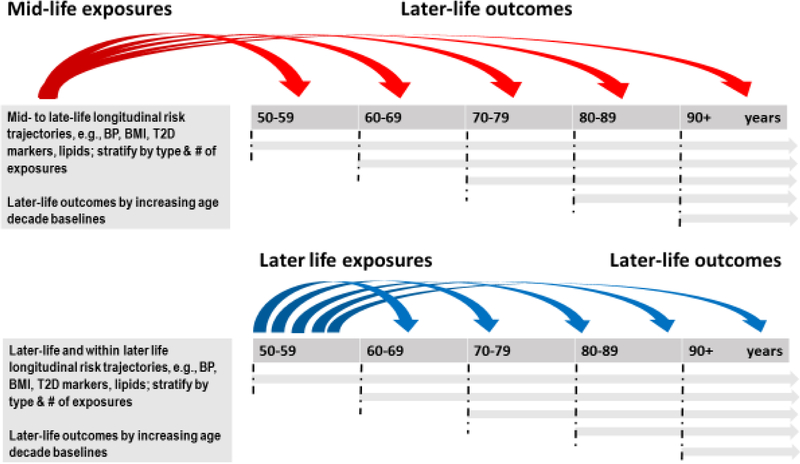
Better mid- and later life identification and characterization of populations at risk for CI and late-onset, sporadic dementias. Life course epidemiology points to the importance of timing. Mid- and late-life exposures in association with CI and LOD differ depending on when an exposure is measured and the length of time between exposure measurement and clinical disease onset. Also important are the trajectories of change in exposures over time and the variability accompanying these exposures.

**Figure 2.**
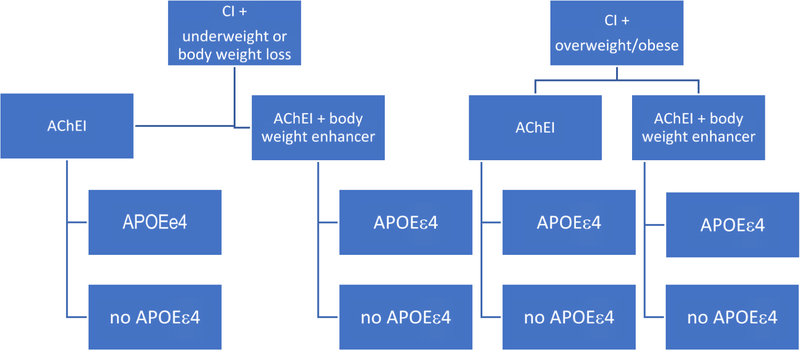
An Example Randomized Controlled Trial Design: The Case of Body Weight and Cognitive Impairments. An example study design is to compare patients with CI who are underweight or experience body weight loss with patients who are overweight or obese. This 18–24 month intervention compares commonly used AChEI, which as a class have been shown to be beneficial for cognition and contribute to body weight loss versus a combined therapy of AChEI plus a body weight enhancer. We hypothesize that patients randomized to AChEI + body weight enhancer will maintain cognitive function and body weight, and have less cognitive decline, and behavioral and psychological symptoms, compared to those not receiving combined therapy.

RCT do not completely address prevention of CI and LOD due to lack of critical evaluation of the observational epidemiology (Table 2, Figure 1). Efforts to facilitate this process include the following. First, longitudinal and RCT data base compilation efforts funded by NIH and other funding agencies e.g., IALSA/Maelstrom (https://www.maelstrom-research.org); STROKOG (https://cheba.unsw.edu.au/group/strokog); GAAIN (https://www.gaain.org) and www.clinicaltrials.gov) provide platforms for in-depth evaluation of the epidemiologic literature. Second, routine monitoring of phenotypic changes with aging should be initiated in Geriatrics, Primary Care and/or Subspecialty clinics to identify at risk subgroups for enrollment in RCTs. Third, duration of exposure(s) required to achieve desired effects observed in the observational epidemiology is ignored in RCTs. Participants enter an RCT with a past. Despite recall bias, in lieu of universal medical records, information should be gathered. Longer-term follow-up of adults with multi-morbidities and who are adherent over time to, for example, antihypertensive agents or antiretroviral therapies, versus those who are not adherent may be invaluable. This is especially important given cardiovascular drug repurposing for LOD prevention. Finally, the concept of Multiple Etiologies Dementias (MED), a diagnostic construct that emerged from the 2016 Alzheimer’s Disease and Related Disorders Summit, opens the door to alternate pathways of CI detection, and lends to exploration and expansion of CI detection methods and locations across a spectrum of vascular and other risk (https://www.ninds.nih.gov/sites/default/files/ADRD2016_summit_council_rpt_508Cpdf.pdf). From the standpoint of MED, the observational epidemiology is on the mark. The observational epidemiology can inform RCT with more rigorous and attentive implementation.

## Figures and Tables

**Table 1.** Issues for Critically Evaluating the Epidemiology of Vascular Exposures Associated with Cognitive Impairments and Late-Onset, Sporadic Dementias.

Definition of the exposures Definition of the outcomes: LOD, LOD subtype, CI Characteristics of the population at risk Age of exposure or onset of outcome Duration of exposure Timing of exposure in relation to outcome Survival time Birth cohort Study design Analysis strategy Competing risks Molecular epidemiology – genes and biomarkers

**Table 2.** Upgrade Overdue. Translating the Observational Late-Onset Dementia Epidemiology to Randomized Clinical Trials.

**Observational Cl & Late-Onset, Sporadic Dementia Epidemiology**	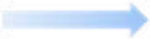	**Randomized Clinical Trials**
Prioritize intermediate phenotypes	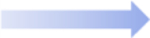	Intermediate phenotypes as primary or secondary outcomes
Wisely select pooled or global replication analyses of existing data to refine linkages between exposure and outcome	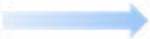	Refine linkages
Stratify by gene variants relevant for pharmacokinetics, metabolic and vascular risk	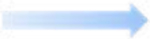	Employ gene stratification
Embrace multi-morbidities (multiple exposures)	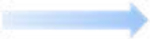	Recruit on the basis of multi-morbidities
Enhance analysis of polypharmacy	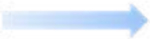	Recruit on the basis of polypharmacy
Trajectories of medication use across populations	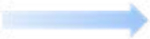	Chronicity of medication use
Renew attention to medication adherence and management on a global scale	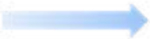	Incorporate adherence patterns in design and data interpretation
Define and consider global health disparities	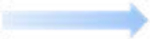	Actively include health disparities samples
More industry partnerships to improve exposures	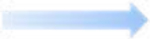	Use better exposures with industry partners
**End result: Observational data linked more directly and efficiently with clinical trial implementation**.		

**Table 3.** Selected Summary of Observational Epidemiology of Body Mass Index, Cognition and Late-Onset, Sporadic Dementias.

**Cross-sectional analyses** Late-life low BMI → prevalent LOD^28^ Late-life higher adiponectin → worse cognition and worse AD brain neuroimaging outcomes^29^ Mid-life higher adiposity → better cognition among a vulnerable sample^30^
**Longitudinal analyses** First Report: Late-life more overweight among women born 1901/02 in their 70s →AD at ≥ 80 years^31^ Higher waist-to-hip ratio → higher LOD risk after 32 years^32^ Declining BMI preceding LOD and with usual aging^8^ 37 year natural history of BMI and LOD^33^ Steeper late-life BMI decline with APOEε4 allele among those with and without LOD^8^ Late-life low BMI → LOD within 5 years^34^ Higher BMI + no APOEε4 → slower AD progression over 1y based on CDR-Sum of boxes^35^ Lower BMI + APOEε4 → faster AD progression over 1y based on CDR-Sum of boxes^35^

**Table 4.** Glucose lowering drugs reported at baseline in the ACCORD Type 2 diabetes trial, mechanisms of action, and potential effects on body weight.

Agent	Examples	Mechanism of Action	Effect on Body Weight
**Meglitinides**	Repaglinide (Prandin)	ATPK inhibitor	Increase
**Alpha-glucosidase inhibitors**	Acarbose, miglitol	Inhibit breakdown of CHOs	No effect
**Sulfonylureas**	Orinase (tolbutamide), tolinase (tolazamide), diabinese (chlorpropamide), glucotrol (glipizide), glucotrol XL, micronase, Diabeta (glyburide), glynase (micronized gly), amaryl (glimepiride)	ATPK inhibitor; Stimulate beta cells in pancreas to increase insulin	Increase
**Biguanides**	metformin	Decrease gluconeogenesis, AMPK activator	Decrease
**Thiazolidinediones**		PPAR-gamma agonists	Increase
**Insulins**	Regular, glargine, NPH, UL, L, Lispro, Aspart	Nutrient sensing hormone	Increase

CHOs=carbohydrates

Medications for ageing-related comorbidities used to treat, for example, Type 2 diabetes, have differential effects on body weight gain and loss and should be considered when monitoring body weight loss in association with the progression of CI and LOD.
